# Laser-Based Characterization and Classification of Functional Alloy Materials (AlCuPbSiSnZn) Using Calibration-Free Laser-Induced Breakdown Spectroscopy and a Laser Ablation Time-of-Flight Mass Spectrometer for Electrotechnical Applications

**DOI:** 10.3390/ma18092092

**Published:** 2025-05-02

**Authors:** Amir Fayyaz, Muhammad Waqas, Kiran Fatima, Kashif Naseem, Haroon Asghar, Rizwan Ahmed, Zeshan Adeel Umar, Muhammad Aslam Baig

**Affiliations:** 1 Atomic and Molecular Physics Laboratory, Department of Physics, Quaid-i-Azam University, Islamabad 45320, Pakistan; 2 National Centre for Physics, Quaid-i-Azam University Campus, Islamabad 45320, Pakistan; 3 Department of Mining and Geological Engineering, The University of Arizona, Tucson, AZ 85719, USA; waqas@arizona.edu; 4 School of Energy and Material Science, Hunan University of Humanities Science and Technology, Loudi 417000, China

**Keywords:** functional materials, laser, plasma, random forest, laser ablation, mass spectrometer

## Abstract

In this paper, we present the analysis of functional alloy samples containing metals aluminum (Al), copper (Cu), lead (Pb), silicon (Si), tin (Sn), and zinc (Zn) using a Q-switched Nd laser operating at a wavelength of 532 nm with a pulse duration of 5 ns. Nine pelletized alloy samples were prepared, each containing varying chemical concentrations (wt.%) of Al, Cu, Pb, Si, Sn, and Zn—elements commonly used in electrotechnical and thermal functional materials. The laser beam is focused on the target surface, and the resulting emission spectrum is captured within the temperature interval of $9.0\times 10^{3}$ to $1.1\times 10^{4}$ K using a set of compact Avantes spectrometers. Each spectrometer is equipped with a linear charged-coupled device (CCD) array set at a 2 μs gate delay for spectrum recording. The quantitative analysis was performed using calibration-free laser-induced breakdown spectroscopy (CF-LIBS) under the assumptions of optically thin plasma and self-absorption-free conditions, as well as local thermodynamic equilibrium (LTE). The net normalized integrated intensities of the selected emission lines were utilized for the analysis. The intensities were normalized by dividing the net integrated intensity of each line by that of the aluminum emission line (Al II) at 281.62 nm. The results obtained using CF-LIBS were compared with those from the laser ablation time-of-flight mass spectrometer (LA-TOF-MS), showing good agreement between the two techniques. Furthermore, a random forest technique (RFT) was employed using LIBS spectral data for sample classification. The RFT technique achieves the highest accuracy of ~98.89% using out-of-bag (OOB) estimation for grouping, while a 10-fold cross-validation technique, implemented for comparison, yields a mean accuracy of ~99.12%. The integrated use of LIBS, LA-TOF-MS, and machine learning (e.g., RFT) enables fast, preparation-free analysis and classification of functional metallic materials, highlighting the synergy between quantitative techniques and data-driven methods.

## 1. Introduction

Metal alloys are used for the fabrication of various packaging materials, airplane parts, vehicles, and construction materials due to their unique properties, such as resistance to rust, high conductivity, non-toxicity, recyclability, flexibility, and reflectivity. These alloys play critical roles in numerous industrial applications, including chemical equipment, aerospace components, electrical conductors, solders, and welding electrodes [1]. Modifications to an alloy’s composition can significantly impact its quality by influencing its physical, chemical, and mechanical properties. For example, metallic alloys comprising Al, Cu, Pb, Si, Sn, and Zn are widely used as functional materials in electrotechnical and thermal applications due to their favorable mechanical, electrical, and corrosion-resistant properties. Therefore, the precise compositional analysis of functional alloy systems is critical for quality control and performance optimization in electrotechnical devices. The exploration and advancement of techniques for precise elemental analysis of metal alloys are not only intriguing but also highly imperative. Various analytical techniques have been employed for compositional analysis, including Rutherford backscattering spectrometry (RBS), energy-dispersive X-ray spectroscopy (EDX), X-ray fluorescence (XRF), inductively coupled plasma mass spectrometry (ICP-MS), inductively coupled plasma-optical emission spectrometry (ICP-OES) [2,3,4,5], and proton-induced X-ray emission (PIXE) [6]. While these methods are generally observed as reliable, they challenge limitations when scaled up for industrial applications. For instance, quantitative XRF results are often inadequate for accurately determining the composition of the metal core [7]. This technique provides only an average elemental composition based on radiation energy and the composition of surface corrosion layers, which penetrate depths from microns to tens of microns [7]. Consequently, XRF typically does not yield direct information about the core composition of metallic objects [7], and its spatial resolution is limited to ~20 μm [8]. Similarly, although ICP-OES and ICP-MS are highly sensitive, these conventional techniques require lengthy sample preparation, involving hazardous chemical solvents and reagents, making them time-consuming and impractical for in-field or remote monitoring applications [9].

Alternatively, laser-induced breakdown spectroscopy (LIBS) is widely used for the analysis of materials by focusing a high-energy laser pulse on the sample, generating a plasma, and analyzing the emitted light to determine the elemental composition of the target, which can be performed in situ, on-site, or remotely depending on the application [10,11,12,13,14,15]. Compared to traditional techniques, LIBS provides several advantages for spectral analysis, including minimal sample preparation, the ability to analyze solid, liquid, or gaseous samples, rapid real-time measurements, the capability for in situ or remote sensing applications, and the detection of a wide range of elements with high sensitivity and spatial resolution [16,17,18]. Quantitative analysis in LIBS can be performed via the calibration curve method (CC-LIBS) [17,18] or the calibration-free approach (CF-LIBS) [11,19,20]. In CC-LIBS, calibration curves are generated by correlating emission line intensities from reference materials with known concentrations, which are then used to quantify unknown samples. While effective for samples with analogous matrices, CC-LIBS is constrained by matrix effects and spectral self-absorption. In contrast, CF-LIBS eliminates the need for external calibration standards and allows for the detection of all elements present, but it requires conditions of optical thinness and local thermodynamic equilibrium (LTE) for accurate plasma characterization.

LIBS combined with machine learning algorithms suggests rapid and innovative approaches to enhance classification accuracy. Different multivariate chemometric algorithms have been employed for classification analysis, such as SVM, ANN, and PLS-DA. The conventional classification techniques have two main drawbacks for example overfitting in SVM and ANN and low prediction accuracy in PLS-DA. Various classification techniques have been established to improve classification accuracy. For instance, the random forest technique (RFT) is an efficient and one of the most effective modern classification algorithms in the machine learning field that relies on multiple classifiers [21]. It can address the issues of low accuracy and overfitting found in traditional classification algorithms. The RFT algorithm is an ensemble method consisting of unpruned classification trees through bootstrap samples of the training data and random feature selection during tree construction. The final category for classification samples can be determined by gathering the estimates from numerous classifiers using a majority vote method. The classification performance of the RFT method can be assessed by measuring the generalization error using N-fold cross-validation.

In this study, we analyze and classify multi-component metallic alloys as potential functional materials using different techniques. We combined CF-LIBS, laser ablation time-of-flight mass spectrometer (LA-TOF-MS), and RFT techniques to rapidly analyze numerous pelletized metal alloy samples. In the first phase, plasma characterization was performed, followed by determining the electron number density and plasma temperature. In the second phase, quantitative analysis of the samples was performed using CF-LIBS. Emission lines of interest were selected, including Al (I) at 309.27 nm, Zn (I) at 577.21 nm, Sn (I) at 452.47 nm, Si (I) at 243.52 nm, Cu (I) at 510.55 nm, and Pb (I) at 373.99 nm. The net (i.e., total intensity minus background) normalized integrated intensities of selected emission lines were utilized for the analysis. The intensities were normalized by dividing with the net integrated intensity of the aluminum emission line (Al II) at 281.62 nm for each sample individually. These lines were chosen for their unsaturated signal intensity, minimal self-absorption, and spectral interferences. The CF-LIBS results were compared with those obtained from LA-TOF-MS, showing good agreement. In the third phase, LIBS spectral data were used in RFT procedures for classification analysis. A total of 400 LIBS spectra were utilized, with 320 spectra designated for the training set and 80 for the testing set. Optimization of two parameters, the number of trees and random variables, was achieved through out-of-bag (OOB) estimation. The training set was used to develop the RFT classification model, while the testing set evaluated its predictive performance based on accuracy, precision, recall, and F1-score. We integrate LIBS with machine learning algorithms to overcome the limitations of conventional elemental analysis. The combination presents a rapid, preparation-free, and robust method for the accurate classification of functional metallic materials.

## 2. Materials and Methodology

The present study follows a comprehensive framework comprising: (i) sample preparation and detailed experimental setups for LIBS and LA-TOF-MS; (ii) spectral preprocessing, normalization, and plasma characterization including temperature, electron number density, LTE, and optically thin condition validation; (iii) an overview and implementation of the Random Forest (RFT) classification technique; (iv) optimization of RFT parameters, classification performance, and validation; (v) quantitative analysis using CF-LIBS and LA-TOF-MS; and (vi) interpretation and discussion of results in the context of prior studies. Each phase is systematically addressed in the following sections.

### 2.1. Sample Preparation

Nine samples, containing different chemical compositions (wt.%) of metals including Al, Cu, Pb, Si, Sn, and Zn, were prepared. Fine powdered pure metals were obtained from Sigma, Aldrich Germany. The purity of the commercial metal powders ranges from 99.5 to 99.9%. These metals were meticulously weighed and then blended in a mortar pestle. The pestle was initially cleansed with purified water, subsequently dried in an electric drying oven set at a temperature between 70 and 80 °C, and finally rinsed with ethanol to eliminate any potential impurities. To ensure precision in measurement, the powders were weighed in a controlled environment using a digital weighing balance enclosed within a glass cage, guarding against fluctuations caused by air drafts, especially crucial for samples with minute amounts of added metals. The accuracy of weight measurements was ensured by using a high-precision analytical balance with an accuracy of ±0.01 mg for small samples (~7 mg) and ±0.001 g for larger samples (~7 g). The resultant metallic blends were then brought together into stainless steel dies and compressed using a hydraulic press to form pressed pellets weighing ~7 g each, with dimensions of 25 mm in diameter and 6 mm in height. Gradual pressure was exerted using the hydraulic press to compact the samples into solid pellets, with a consistent pressure of 50 MPa applied for 20 min. Table 1 illustrates the chemical composition of different metals in the alloy samples.

### 2.2. LIBS Experimental Methods

**Figure 1:**
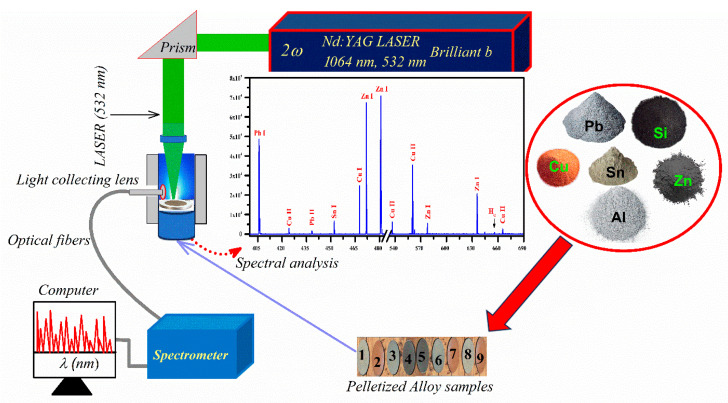
Schematic diagram of custom-built bench-top LIBS experimental system.

In this study, the custom-built bench-top experimental system is utilized to record the optical spectra of the laser-induced plasma as detailed in our earlier reported work [22,23]. The schematic diagram of the LIBS system is shown in Figure 1. To summarize, a high-power Q-switched Nd: YAG laser (Quantal, France, Brilliant-B) with a 5 ns pulse duration and a 10 Hz repetition rate was utilized to generate analytical plasma. The analytical plasma, characterized by high temperature (~$9.0\times 10^{3}$ to $1.1\times 10^{4}$ K) and pressure, emits light as excited atoms and ions return to their ground states. The pulse Nd: YAG laser can generate pulses with energies of ~850 mJ at wavelength 1064 nm and ~400 mJ at wavelength 532 nm, with energy measurement conducted using a Nova-Quantel energy meter (France). The laser beam was focused onto a micron-sized spot with a quartz convex lens having a 20 cm focal length. The size of the focused laser spot was determined by employing thermal paper placed ~0.05 cm before the lens’s focal length, with subsequent diameter measurement using an optical microscope at a laser pulse of ~100.0 ± 0.3 mJ. The resulting focused beam diameter at the target was ~50 μm, with a corresponding laser fluence of about 10 to 15 Jcm^−2^.

The samples were mounted on a manually rotating stand to avoid the creation of intense cracks, ensuring a fresh surface for each laser shot. Additionally, to prevent air breakdown in front of the sample, the space between the sample and lens stand was maintained shorter than the length focal spot. Light emitted by the analytical plasma during cooling was captured using an optical fiber accompanied by a collimating lens positioned perpendicular to the laser beam. Optical fiber (~600 µm core diameter) with a high hydroxyl (OH) fused silica content was used for the efficient transmission of UV light. In our experiment, the collimating lens, with a field of view ranging from 0° to 45°, was positioned to capture a significant portion of the plasma plume. The distance from the plasma to the lens was 10 mm, ensuring optimal light collection without risking damage to the optical components. The light signal from plasma was then recorded using six compact miniature spectrometers (Holland, Avantes, Apeldoorn, Netherlands) each equipped with a 10 µm slit width, covering an optical region from 220 to 970 nm. The instrumental width of the spectrometer in the range of 550 to 690 nm was determined from the observed zinc (I) emission line at 577.21 nm as 0.06 ± 0.01 nm. Gate delay (t_g_) and integration time (t_i_) were set at 2 μs and 10 ms, respectively, to ensure unsaturated emission spectra. To reduce signal variation, 30 single-shot spectra were acquired and averaged for each sample. The sample pellets were moved manually on a horizontal flat surface in a nearly random pattern to ensure fresh surface spots were sampled, while the vertical position of the sample relative to the focusing lens remained constant.

### 2.3. LA-TOF-MS Spectrometric Studies

**Figure 2:**
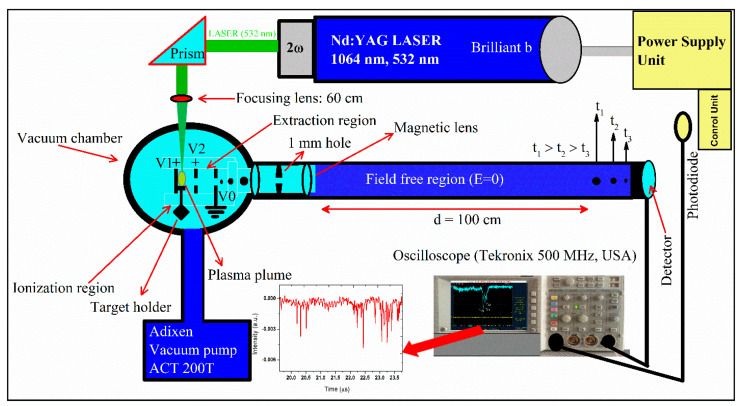
Schematic diagram of custom-built LA-TOF-MS experimental system.

In this study, the compositional analysis was also achieved using the LA-TOF-MS technique following our experimental setup as previously reported [24,25]. A schematic illustration of the experimental arrangement is provided in Figure 2. In short, the system was locally designed and constructed on the theory of Wiley–McLaren-type equipment. The experimental setup contains a stainless steel vacuum chamber with a 300 mm diameter, the ionization region, the extraction region, and an 8 cm × 100 cm ion-beam flight tube. The vacuum of the whole system was kept at ~10^−6^ mbar through a turbomolecular pump supported by a motorized pump during the experimentation. Three metal cathodes were utilized in this equipment, each with a circular shape with a diameter of 30 mm, mutually separated by a distance of 15 mm. Two of the electrodes hold a 10 mm opening window in the center which is covered with a fine tungsten mesh.

The target sample placed under a high vacuum was ablated with a few mJ laser pulses from the same LIBS laser system. The laser beam was focused using a quartz lens having a focal length of 60 cm. The calculated focused beam spot radius at the sample surface was about 0.5 (±0.1) mm. The emitted ions from the ablated target sample were accelerated toward the detector using an electric field and focused on the detector using a cylindrical shape magnetic filter. The magnetic filter was positioned in the path of the ion beam near the metallic electrodes. A non-magnetic aluminum piece of disc shape having a 1.5 mm opening at its center was inserted into the magnetic lens just to cut the dispersed beam portion. The ion signal was registered using the channeltron detector (Galileo, USA) functioning at about a DC voltage of 2100 to 3000 V. The channeltron detector was synchronized with an oscilloscope (Tektronix, USA) with a 500 MHz digital storing capacity to analyze the ion signal. The oscilloscope was stimulated by a fast photodiode by a fraction of the laser beam.

### 2.4. Random Forest Technique

Random forest technique (RFT) is a supervised machine learning procedure used for classification, regression, and other tasks. It operates by constructing a multitude of decision trees during training and outputting the class that is the mode of the classes (classification) or mean prediction (regression) of the individual trees. The method combines the predictions of multiple trees to improve the overall performance and reduce the risk of overfitting. By exploiting the “wisdom of the crowd”, random forest often achieves high accuracy and stability, making it a go-to choice for many practical machine-learning applications.

**Figure 3:**
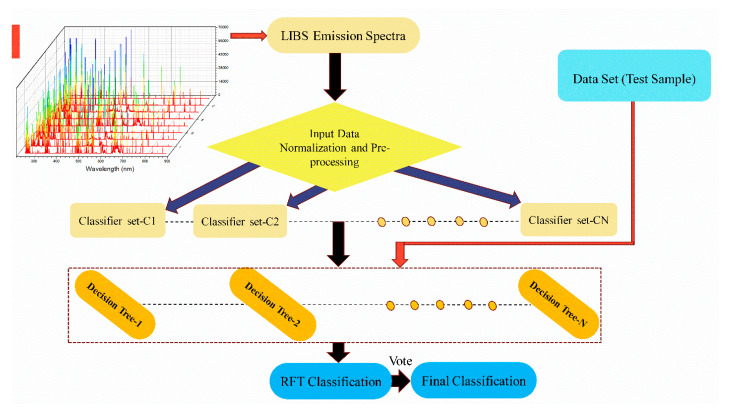
Schematic diagram of the RFT process.

**Figure 4:**
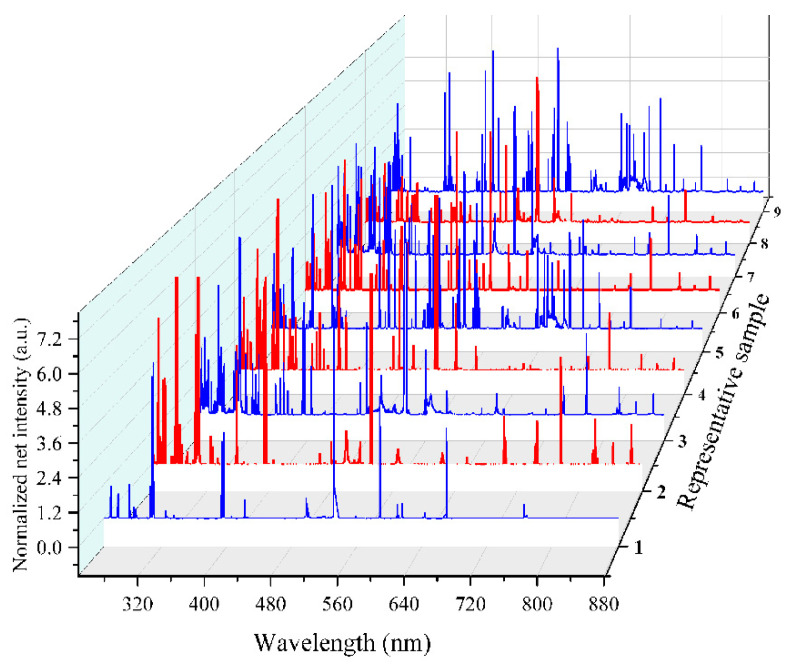
The normalized representative LIBS emission spectra of the pelletized metal alloy samples.

In the RFT methodology, LIBS emission spectra featuring the characteristic light emitted by a sample upon laser ablation are acquired. These spectra are then normalized and preprocessed to remove noise and standardize the data, ensuring consistent input for the model. Following preprocessing, the data are fed into multiple classification trees formed within an RFT framework. Each tree in the random forest independently classifies the input spectra. The final prediction is determined by a simple majority voting system, where the most frequent class prediction among all individual trees is chosen as the overall result. This ensemble approach enhances the accuracy and reliability of the classification compared to a single decision tree (see Figure 3). Figure 4 displays the representative LIBS emission spectra of nine pelletized metal alloy samples (such as AlCuPbSiSnZn) featuring varying chemical concentrations and normalized for spectral intensity with the standard deviation. The classification of these samples was performed using RFT analysis executed in MATLAB (R2016a).

In this study, the following steps were assumed to perform RFT analysis: (i) From a given training dataset ***D***, LIBS emission spectra with **n** samples, and **m** bootstrap samples *D*_1_, *D*_2_, ……, *D_m_* are created by random sampling with replacement from ***D*** spectra. Each bootstrap sample D_i_ contains **n** samples. (ii) For each bootstrap sample *D_i_*, we trained a decision tree *T_i_*. During the construction of each tree, at each node, a random subset of features *F_i_* of size ***m****_try_* from the total *P* features (wavelengths) was selected. For classification tasks, ***m****_try_* is taken as $\sqrt{P}$ along with ***m****_try_ =* P/3 for regression tasks. (iii) For each tree *T_i_*, the samples not included in the bootstrap sample *D_i_* are considered out-of-bag (OOB) samples. These OOB samples were used to estimate the model’s performance. The error rate was calculated by predicting the OOB samples using the trees that did not see these samples during training. The overall OOB error was taken as the average error for all OOB samples across all trees, providing an unbiased estimate of the model’s performance. (iv) Each tree *T_i_* has cast a vote for a class, and the final prediction for sample *x* is determined by the majority vote among the trees for classification. For example, $\hat{y}=mode\{T_{1}\left(x\right),\;T_{2}\left(x\right),\ldots \ldots ,\;T_{m}\left(x\right)\}$ represents the predictions made by individual decision trees in the forest, where *T_i_(x)* is the prediction of the *i^th^* tree for an input *x*. The mode function takes all these predictions and returns the most frequent one (the majority vote), which is the final output $\hat{y}$ for classification tasks. The final prediction for a sample *x* is taken as the average of the predictions from all trees for regression such as $\hat{y}=\frac{1}{m}\sum_{i=1}^{m}T_{i}\left(x\right)$. The OOB error is estimated for classification using the following relation:

(1) $${\mathrm{Error}}^{\left(\mathrm{OOB}\right)}=(\frac{1}{n}\sum_{i=1}^{n}L(\hat{y_{i}},\;{\hat{y}}_{OOB,i})$$

Here, $L(\hat{y_{i}},\;{\hat{y}}_{OOB,i})$ denotes the indicator function and *n* characterizes the total number (#) of OOB samples. The classification performance of the RFT process with the OOB method is estimated by measuring recall and precision using the following coupled equations.

(2) $$Recall\;\left(100\%\right)=TP/\left(TP+FN\right)$$

(3) $$Precision\;\left(100\%\right)=TP/\left(TP+FP\right)$$

Here, *FP* refers to the original count of functional points mistakenly identified as constant despite being variable after an event. *TP* represents the original count of functional points that correctly remain constant following the event. *FN* denotes the original count of functional points that, though remaining constant post-event, are incorrectly identified as variable. The OOB estimate is derived from roughly one-third of the samples not included in the training set and is utilized for testing during the training process.

The bootstrapping (Bagging method) in the RFT method is used to reduce variance within a noisy data set. The bootstrap data are used for the feature selection and to estimate the classification performance of the RFT model. For any given training set, the forest did not utilize individual votes from all decision trees based on their respective training sets. Instead, it relied on the majority vote for classification results. All training samples were utilized to determine classification accuracy, which is defined as the ratio of correctly classified samples to the total number of samples. This classification accuracy reflects the performance of the classifier. Bootstrap sampling was used to create different training subsets for *n* samples from the original dataset. Approximately 30 to 37% of the initial dataset samples were eliminated in the bootstrapped subsets using Equation (4) [26].

(4) $$\underset{n\to \infty }{\mathrm{Lim}}{\left(1-\frac{1}{n}\right)}^{n}\to 2.72^{-1}\sim 36.8\%$$

Each sample from the original dataset has a 1n probability of being selected. Over multiple trials (as *n* grows large), the probability of a sample not being selected in any trial converges to e^−1^ ≈0.368. The 36.8% result holds in the theoretical limit (or asymptotic probability) where *n*→∞. When *n* = 9, the actual result is lower, specifically 34.6%. This difference arises because the theoretical value (e^−1^) is the asymptotic limit, while *n* = 9 represents a finite case. For finite *n*, the actual probability needs to be computed directly, as was carried out for *n* = 9, yielding 34.6%.

## 3. Results

### 3.1. Analytical Plasma and Spectral Analysis

When the laser pulse focuses on the sample surface, it creates an analytical plasma, which expands outward from the sample. Within a few microseconds, this analytical plasma begins to cool. During this cooling process, emissions occur that carry the unique characteristics of the elements within the sample. These emissions, or spectra, are captured and analyzed to collect information about both the prominent and minor elements contained within the sample. In this study, samples are examined under atmospheric pressure conditions, enabling the confinement of plasma and the observation of spectral lines associated with hydrogen (H), oxygen (O), and nitrogen (N). These spectral lines serve as valuable indicators for analytical plasma diagnostics. Spectra were collected from the analytical plasma under optimized experimental conditions, including a laser energy of 100.0 ± 0.3 mJ, corresponding to a laser fluence of 10 to 15 Jcm^−2^, with t_g_ and t_i_ times set at 2 μs and 10 ms, respectively. All emitted spectra from samples (1 to 9) were acquired. However, sample 6 contained an appropriate concentration of wt.% of elements Zn, Sn, Si, Cu, and Pb, making it the selected representative sample.

**Figure 5:**
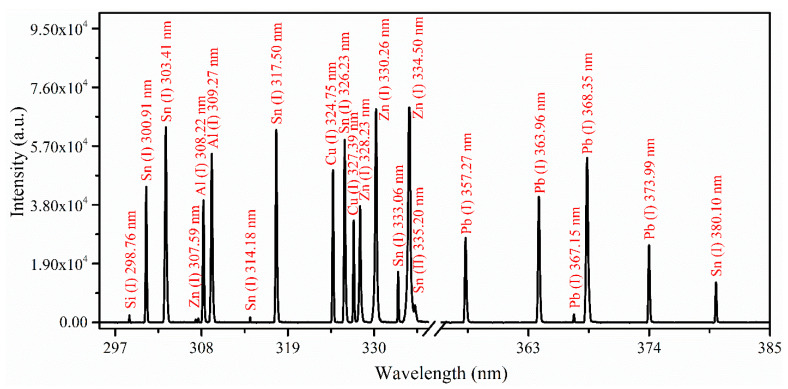
The spectrum of sample 6 recorded using optimized laser energy of ~100.0 ± 0.3 mJ with a corresponding laser fluence of ~10 to 15 Jcm^−2^, displays the emission lines of Si, Sn, Zn, Al, Cu, and Pb across the wavelength range from 296 to 385 nm.

Figure 5 displays the emission spectrum of sample 6, spanning wavelengths from 296 to 385 nm. Each spectral line is identified with its transition central wavelength. Significant spectral lines from Si, Sn, Zn, Al, Cu, and Pb were identified within this optical range. The strong emission line of Al (I) at a wavelength of 308.22 nm, arising from the electronic transition of 3*d* ^2^*D*_3/2_ *→* 3*p* ^2^*P*_1/2_, is detected among all observed spectral lines of the elements. The estimated relative intensity ratio between the two emission lines of Al (I) at 308.22 nm and 309.27 nm from the sample 6 spectrum is in agreement with values from the NIST database [27]. The experimentally observed intensity ratio was 1.46, while the calculated ratio, based on spectroscopic parameters—wavelength λ (308.22 nm, 309.27 nm), transition probability A_k_g_k_ (2.35 × 10^8^, 4.37 × 10^8^ s^−1^), upper energy level E_k_ (4.02148, 4.02165 eV), and electron temperature T_e_ (0.89 eV for sample 6)—was 1.84, both within a 20% relative error. In this spectral region, the resonance spectral line of Zn (I) at 307.59 nm is also observed, corresponding to the electronic transition from the 4*s*4*p* ^3^*P*_1_ state to the 4*s*^2 1^*S*_0_ ground state. Additionally, a characteristic doublet of Cu (I) emission lines at 327.39 nm and 324.75 nm appear within the UV spectral range. These emission lines result from electronic transitions between discrete energy levels in copper atoms, specifically from the 4*p* ^2^*P*_3/2,1/2_ states to the 4*s* ^2^*S*_1/2_ ground state.

**Figure 6:**
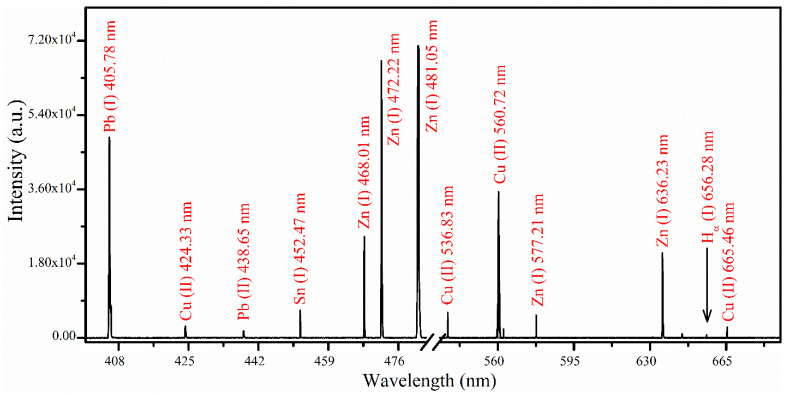
The spectrum of sample 6 recorded using an optimized laser energy of ~100.0 ± 0.3 mJ, with t_g_ and t_i_ times configured at 2 μs and 10 ms, respectively. The spectrum shows emission lines of Pb, Cu, Sn, Zn, and H across the wavelength range from 398 to 689 nm.

**Figure 7:**
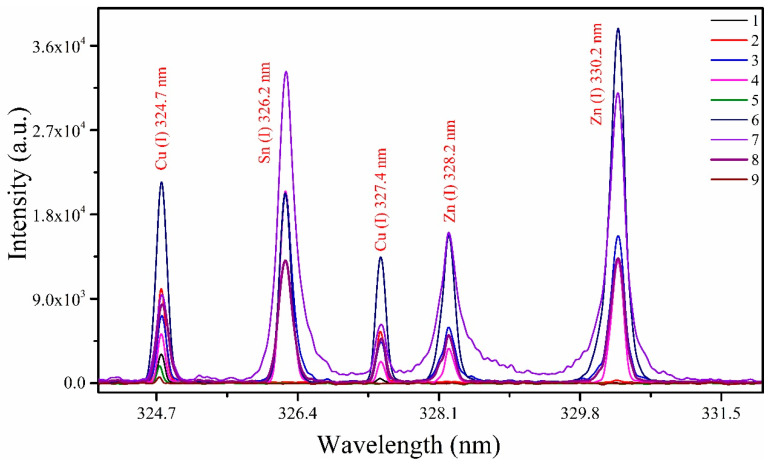
The stacked emission lines exhibit variations in intensity for Cu, Sn, and Zn under identical experimental conditions, including a laser energy of ~100.0 ± 0.3 mJ, corresponding to a laser fluence of roughly 10 to 15 Jcm^−2^, t_g_, and t_i_ times set at 2 μs and 10 ms, respectively, and a focused beam diameter at the target of ~50 μm. These variations were observed within the wavelength range of 324 to 332 nm.

Figure 6 shows the emission spectrum of the same representative sample 6 in the range from 398 to 689 nm. Various spectral lines from Pb, Cu, Sn, H, and Zn were identified within this optical range. The intense spectral line of Pb (I) at a wavelength of 405.78 nm has been successfully identified. A cluster of emission lines from Zn (I) at wavelengths of 468.01 nm, 472.22 nm, and 481.05 nm arise from transitions originating from the same upper level (5*s* ^3^*S*_1_) to lower levels (4*p* ^3^*P*_0,1,2_), respectively, exhibiting an elevated intensity ratio. Furthermore, the presence of hydrogen *H_α_* (I) at 656.28 nm has been detected, attributed to the contribution of the air environment to the sample plasma. In Figure 7, comparative analytical spectra of all samples (1 to 9) under identical optimized experimental parameters are presented within the wavelength range from 324 to 332 nm. The variations in the intensity of emission lines at wavelengths 324.75 nm, 327.4 nm for Cu (I), 326.2 nm for Sn (I), and 328.2 nm, as well as 330.2 nm for Zn (I), were examined. Notably, emission lines corresponding to Sn (I) at 326.2 nm and Zn (I) at 328.2 nm and 330.2 nm are absent in samples (1 and 2) within this spectral range, likely due to the relatively low concentrations of these metals.

**Figure 8:**
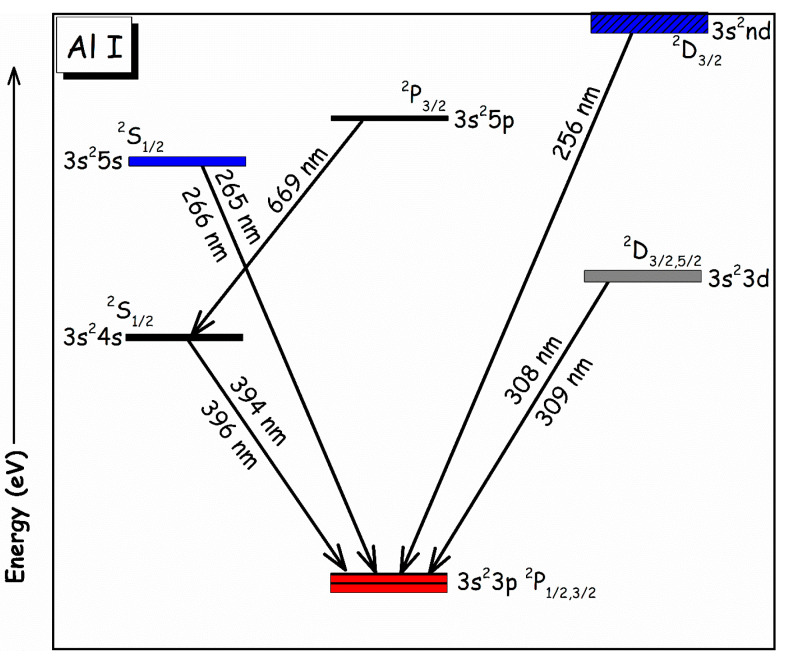
The energy-level diagram with the ground-state electron configuration [Ne] 3s^2^3p^1^ and the corresponding ground levels ^2^P_1/2_ and ^2^P_3/2_, for illustrating various transitions in laser-produced alloy plasma.

Aluminum is present in sample 6 at a high weight percentage, ~55%, compared to other elements such as Pb, Si, Cu, Sn, and Zn. As a result, well-isolated aluminum emission structures were captured in the 250–700 nm optical region. These emission lines include transitions such as 256.79 nm and 308.22 nm (^2^*D*_3/2_ *→* ^2^*P*_1/2_), 266.04 nm and 396.15 nm (^2^*S*_1/2_ *→* ^2^*P*_3/2_), 265.25 nm and 394.40 nm (^2^*S*_1/2_ *→* ^2^*P*_1/2_), 309.27 nm (^2^*D*_5/2_ *→* ^2^*P*_3/2_), and 669.60 nm (^2^*P*_3/2_ *→* ^2^*S*_1/2_). Figure 8 presents an energy-level diagram of the excited states for these aluminum emission lines. These transitions can be utilized for plasma characterization in aluminum plasmas. The lines cover a broad spectral range and exhibit strong intensities, ensuring prominent signals and leveraging the spectrometer’s sensitivity. The wide range of upper-level energies (25,347 cm^−1^ to 40,277 cm^−1^) is useful for plasma characterization, improving the accuracy of measurements. Higher upper-level energies reduce self-absorption effects, while the element-specific properties of aluminum lines ensure accurate identification and minimize spectral overlap. The diverse upper-level energies allow precise calculations of population distributions, reducing systematic errors. This ultimately results in reliable measurements for aluminum-based alloy samples. In this study, LIBS parameters including laser pulse energy, delay time (t_g_), and integration time (t_i_) were optimized to reduce self-absorption phenomena, thereby ensuring unsaturated signals. During measurements, we varied the laser pulse energy in the range of 80–150 mJ to assess its impact on emission intensity and signal-to-noise ratio. The optimal pulse energy for maximizing the detection of the targeted emission lines (Al (I) at 309.27, Zn (I) at 334.50, Sn (I) at 283.99, Si (I) at 288.16, Cu (I) at 510.55, and Pb (I) at 405.78 nm) was found to be ~100 mJ. In addition to pulse energy, we also optimized the delay time and integration time for signal acquisition. For our study, the delay time was adjusted between 1.5 µs and 3 µs, with 2 µs providing the best balance between minimizing continuum emission and maximizing signal strength. Similarly, we experimented with integration time ranging from 5 ms to 15 ms, eventually selecting 10 ms as the optimal value for capturing the emission lines without compromising temporal resolution.

### 3.2. Analytical Plasma Characterization

In LIBS experiments conducted in the air, accurate plasma characterization is often complicated by the self-absorption of spectral lines and the spatial inhomogeneity of the plasma plume. Several methods have been proposed to minimize the effects of self-absorption, including automated recursive algorithms based on the curve of growth for correcting the intensities of self-absorbed lines [28]. While these methods are effective, they tend to be computationally intensive and time-consuming, which can limit their applications in routine analyses. In addition to self-absorption, spectral interference presents another significant challenge in the analysis of complex, multi-element samples. For robust Boltzmann and Saha–Boltzmann temperature plots, as well as for accurate CF-LIBS analysis, it is advisable to include multiple emission lines to enhance reliability. In this study, we assumed plasma homogeneity as a simplifying condition. To account for potential self-absorption effects, all analytical lines included in the CF-LIBS analysis and temperature Boltzmann plot calculations [29] were carefully evaluated. The integrated intensity of an emitted spectral line from an optically thick plasma, derived from the radiative transfer equation, can be expressed as [28,30]:

(5) $$I_{\lambda }=R_{Inst}I_{P}{(\lambda }_{0})\int_{0}^{\infty }(1-e^{-\nu (\lambda )})d\lambda$$

where $R_{Inst}$ is the instrumental factor, $\lambda_{0}$ is central wavelength [28], ν(λ) corresponds to optical depth (dimensionless), and $I_{P}{(\lambda }_{0})$ is the Planck distribution for blackbody radiation and is defined as;

(6) $$I_{P}{(\lambda }_{0})=\frac{2hc}{\lambda_{0}^{3}}\left(\frac{e^{\frac{∆E}{k_{B}T_{e}}}}{1-e^{\frac{∆E}{k_{B}T_{e}}}}\right)$$

where *c* is the speed of light, *k_B_* is Boltzmann’s constant, *h* is Planck’s constant, ∆E is the transition energy difference between spectral levels, and *T_e_* is the plasma temperature. For a homogeneous plasma in LTE, the optical depth ν(λ) can be defined as [28,30];

(7) $$\nu \left(\lambda \right)=k_{i}V(\lambda )Nl$$

where $k_{i}=\frac{e^{2}\lambda_{0}^{2}}{4\varepsilon_{0}mc}f_{ik}\frac{g_{i}e^{-\frac{E_{i}}{k_{B}T_{e}}}}{Z\left(T_{e}\right)}(1-e^{-\frac{∆E}{k_{B}T_{e}}})$ is a coefficient that depends on spectroscopic parameters; *l* is an absorption path length (cm); $f_{ik}$ is the oscillator strength of the transition; $g_{i}$ is the degeneracy of lower energy level; V(λ) represents the normalized Voigt line profile, which is a function of the half-widths of the Doppler $\left(∆\lambda_{D}\right)$, natural $\left(∆\lambda_{N}\right)$, and Lorentzian $\left(∆\lambda_{L}\right)$ line profiles; λ is the wavelength of the emission line; *N* is the number density; *e* is the charge on an electron; $\varepsilon_{0}$ is the absolute permittivity; and *Z*(*T*) is the temperature-dependent partition function. The natural half-width $\left(∆\lambda_{N}\right)$ is lesser compared to the Lorentzian and can be ignored. Self-absorption impacts the shape of certain emission lines, particularly those that are optically thick. It reduces the intensity of the spectral lines and increases the FWHM. This effect is more pronounced in intense emission lines compared to weaker ones, especially at higher species concentrations. Interestingly, in this study, weak emission lines exhibit the lowest $k_{i}$ values ranging from $0.8-13\times 10^{-30}m^{3}$, whereas intense lines show the highest values, exceeding $8\times 10^{-29}m^{3}$. As the $k_{i}$ coefficient increases, the optical depth also increases, as indicated by Equation (7), which confirms that these lines exhibit self-absorption. The self-absorption coefficient (SA) is defined as the ratio of the experimentally estimated peak height of the spectral line to its theoretical peak height in the absence of self-absorption. Assuming a homogeneous plasma in LTE, SA can be derived from the optical depth of the spectral line within the limits 0 to λ0±∆λL2 as [31,32].

(8) $$SA\approx 1-\left(1-e^{-\nu (\lambda )}\right)/\nu (\lambda )$$

For optically thin lines, we assumed a *SA* of zero, implying no self-absorption. As self-absorption increases, the coefficient approaches 1 for a completely self-absorbed line. It is noteworthy that self-absorption effects become significant as ν(λ) → 1, at which point *SA* can reach values of ~0.36 or higher. In our analysis, we estimated the average self-absorption coefficients for all selected emission lines with lower $k_{i}$ values, including both weak and resonant emission lines. The results indicated that the average *SA* values for selected emission lines were ~≤0.02 for all samples analyzed. This low self-absorption coefficient suggests that the self-absorption effect was sufficiently minimal and did not significantly impact the reliability of our analysis. Therefore, we assumed that the self-absorption effect could be neglected for the present work.

Numerous methods have been proposed for determining plasma temperature, including techniques based on absolute or relative line intensities such as line pair ratios or Boltzmann plots, the line-to-continuum intensity ratio, and others. These methods are typically applied to laser-induced plasmas with temperatures in the range of a few thousand Kelvin up to ~$2\times 10^{4}$ K, depending on experimental conditions. In the present work, the Boltzmann plot method is employed to determine the plasma temperature [11,29]. The accuracy and precision of the temperature measurements primarily depend on spectral lines of neutral and singly ionized species characterized by excitation energy differences in their upper levels. Considering the level population follows a Boltzmann distribution, the integrated line intensity, representing the number of transitions per unit volume per unit time for a transition between the upper-level *k* and the lower-level *i*, is expressed as [11]:

(9) $$I_{ki}=A_{ki}\frac{g_{k}}{\lambda_{ki}\;Z(T)}n_{k}^{s}exp(-\frac{E_{k}}{k_{B}T_{e}})$$

where *A_ki_* and *λ_ki_* are the transition probability and the wavelength, respectively, $g_{k}$ is the statistical weight for the level (2*J_k_* + 1), *Z*(*T*) is the partition function, $n_{k}^{s}$ signifies the total population density for elements *s*, and *E_k_* (eV) is the upper-level energy. Considering the plasma is in LTE and optically thin, the plasma temperature $T_{e}$ for the observed population distribution can be determined using the intensities emitted from multiple excited levels. By linearizing Equation (9), the well-known Boltzmann plot relation can be obtained.

(10) $$\ln\frac{\lambda_{ki}I_{ki}}{A_{ki}g_{k}}=-\frac{E_{k}}{k_{B}T_{e}}+\ln(\frac{n_{k}^{s}}{Z\left(T\right)})$$

**Figure 9:**
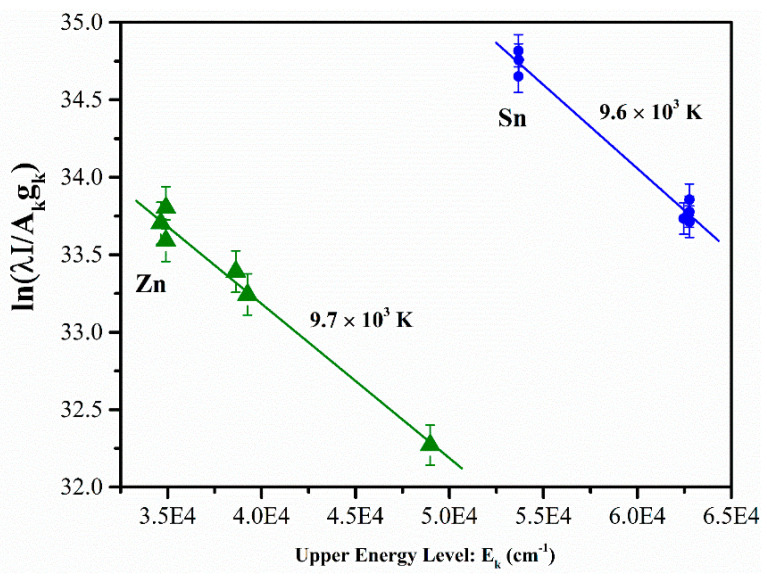
Boltzmann plots of the tin and zinc spectral lines observed in the LIBS spectrum of pelletized alloy sample 6.

A plot of the left-hand side of the relation (10) versus *E_k_* yields a slope of −1/$k_{B}T_{e}$. Consequently, the plasma temperature can be determined through linear regression without requiring prior knowledge of $n_{k}^{s}$ or *Z*(*T*). In this study, we constructed Boltzmann plots using various emission lines of Sn (I) and Zn (I). The selected emission lines included transition central wavelengths for Sn (I) at 300.91, 303.41, 314.18, 333.06, 380.10, and 452.47 nm, as well as for Zn (I) at 328.23, 330.25, 334.50, 468.01, 472.21, 481.05, 577.21, and 636.23 nm. Boltzmann plots were built using the integrated intensity of the emission peaks with normalization. The integrated intensities were normalized by dividing with the integrated intensity of the Al (II) at 281.62 nm. The concentration of aluminum in different samples is not constant. However, we normalized the integrated intensities of the emission lines to the intensity of the Al (II) at 281.62 nm for each sample separately. Time-integrated LIBS emission spectra were used for *T_e_* calculations. However, the *T_e_* of the transient plasma strongly depends on time (delay, integration). The obtained *T_e_* values are, therefore, rough estimates and temporally averaged. The Boltzmann plots using the Sn (I) and Zn (I) emission lines under optimized conditions for sample 6 are shown in Figure 9. Spectroscopic parameters such as the transition probability of various electronic transitions, weighting coefficients, and upper-level transition energies were obtained from the NIST database [27] as shown in Table 2. The data points on these plots exhibited a scattering behavior with an average coefficient of determination (*R*^2^) of ~0.998. Using the slopes of the Boltzmann plots, we estimated the plasma temperature for sample 6 to be $9.7\times 10^{3}$ K based on Zn spectral lines and $9.6\times 10^{3}$ K is based on Sn spectral lines. The error bars on the y-axis (~±0.3) represent the standard deviation in the intensity measurements of the selected emission lines. The calculated electron plasma temperature across all samples ranged from $9.0\times 10^{3}$ to $1.1\times 10^{4}$ K, with an uncertainty of ~±3.5%. The uncertainty of the *T_e_* was derived from the uncertainty in the slope of the Boltzmann fit. Initially, we calculated the uncertainty using the standard deviation of repeated measurements; however, upon further evaluation, we recognized that this method did not provide an accurate estimate of *T_e_*. We have now adopted the standard practice of determining the uncertainty in *T_e_* from the statistical uncertainty of the slope in the Boltzmann plot. This change has resulted in a significant decrease in the estimated uncertainty of *T_e_* from 5% to 3.5%, reflecting a more precise determination of temperature.

Another crucial parameter in characterizing laser-induced plasma is electron number density (*N_e_*), representing the concentration of free electrons within the plasma volume. This metric is vital for recognizing the behavior and interactions of the plasma. Electron number density advances insights into the plasma’s overall charge neutrality, its responsiveness to external fields, and its suitability for various applications, including laser-matter interaction studies, fusion research, and plasma diagnostics. To estimate *N_e_*, one can employ spectral lines of constituent elements that are stark-broadened, well-isolated, and free from self-absorption effects. Among these lines, the stark-broadened *H_α_* emission line at 656.28 nm is preferred due to its minimal self-absorption and high signal-to-noise ratio. Hence, in this study, the *H_α_* spectral line from time-integrated LIBS spectra was used for *N_e_* calculation. The *H_α_* line was utilized to accurately determine electron number density and minimize errors due to self-absorption. However, *N_e_* of the transient plasma strongly depends on time (delay, integration). The obtained *N_e_* values are, therefore, rough estimates and temporally averaged. The formula employed for calculating electron number density is taken from Konjević et al. [33].

(11) $${H_{\alpha }:\;N}_{e}={\left(\frac{{∆\lambda }_{FWHM}}{1.098}\right)}^{1.47135}*10^{17}{\mathrm{cm}}^{-3}$$

**Figure 10:**
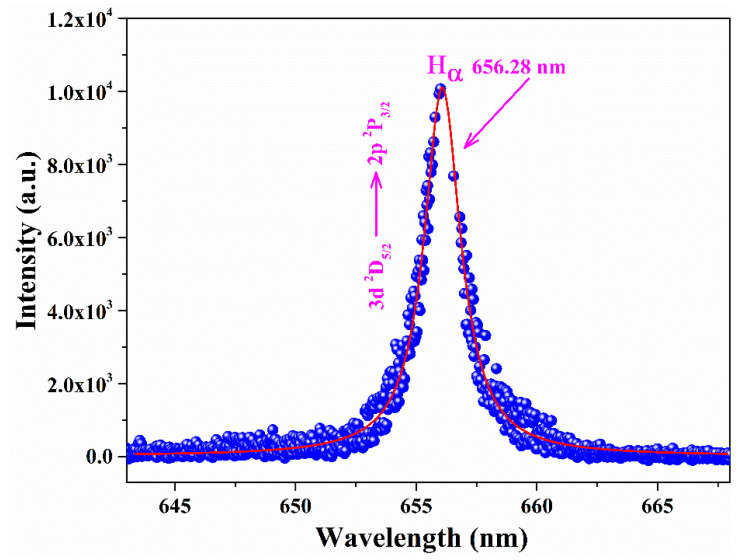
Voigt profile of the H_α_ emission line at 656.28 nm for sample 6.

The *H_α_* line profile with Voigt fitting for sample 6 is shown in Figure 10. The full width at half-maximum (FWHM) of the *H_α_* line at 656.28 nm was determined by de-convoluting the observed line profile into a Voigt profile, which accounts for instrumental width, Doppler width, and Stark broadening. The instrumental width of the spectrometer from 550 to 690 nm is ~0.06 (±0.01) nm, while the estimated Doppler width is about 0.005 nm. The electron number density for each sample was computed using the FWHM of the H_α_ line at 656.28 nm. The estimated electron number density for all samples falls within the range of (0.9–2.1) ± 0.5 × 10^17^ cm^−3^.

In CF-LIBS, achieving optically thin and LTE conditions is crucial for accurate elemental analysis. LTE ensures that the plasma’s excitation temperatures align with the local temperature, simplifying the interpretation of spectral line intensities and enabling reliable quantitative analysis. Optically thin conditions confirm that emitted photons escape the plasma without significant absorption or scattering, allowing for up-front interpretation of the emitted spectra and direct correlation between spectral line intensities and elemental concentrations in the sample. Together, LTE and optically thin conditions in CF-LIBS enable precise elemental analysis, vital for various scientific and industrial applications. To determine whether the plasma is optically thin, the ratio of experimental line intensities was compared with the ratio of the calculated line intensities using the following relation [17,34].

(12) $$\frac{I_{ki}}{I_{nm}}=\left(\frac{\lambda_{nm}}{\lambda_{ki}}\right)\left(\frac{A_{ki}}{A_{nm}}\right)\left(\frac{g_{k}}{g_{i}}\right)Exp\left(-\frac{E_{k}-E_{n}}{k_{B}T_{e}}\right)$$

where *I_ki_* and *I_nm_* represent the observed spectral line intensities at wavelengths *λ_ki_* and *λ_nm_*, respectively. *A_ki_* and *A_nm_* are the transition probabilities, while $g_{k}$ and $g_{n}$ are the statistical weights of the upper levels. In addition, *k_B_* is the Boltzmann constant, and $T_{e}$ denotes the excitation temperature. All spectroscopic data, including statistical weights, transition probabilities, upper-level energies, and central wavelengths for the emission lines of Pb, Si, and Zn, are presented in Table 3, demonstrating consistency between calculated and observed intensity ratios. Emission lines with identical or closely spaced upper level energies were selected to minimize sensitivity to temperature variations. The intensity ratios between the empirically measured emission lines and the values derived from atomic parameters show good agreement, within 10% relative error. This suggests that the plasma meets the criteria for optical thinness.

The second condition to be satisfied is LTE, which is determined by the McWhirter Criterion [35]. This criterion establishes the minimum electron number density needed for LTE by comparing the electron collision frequency with the frequency of radiative transitions between energy levels. According to the McWhirter Criterion, if the electron collision frequency significantly dominates the frequency of radiative transitions, LTE can be assumed. So, meeting this condition indicates that collisional processes predominate over radiative processes, thereby satisfying LTE within the plasma. In this study, the electron number densities were estimated to range from 10^13^ to 10^14^ cm^−3^, specifically lower compared to those calculated using Stark broadening parameters, which fall within the range of (0.9–2.1) × 10^17^ cm^−3^. Consequently, it is evident that the laser-induced plasma is in LTE.

### 3.3. RFT Parameter Optimization

**Figure 11:**
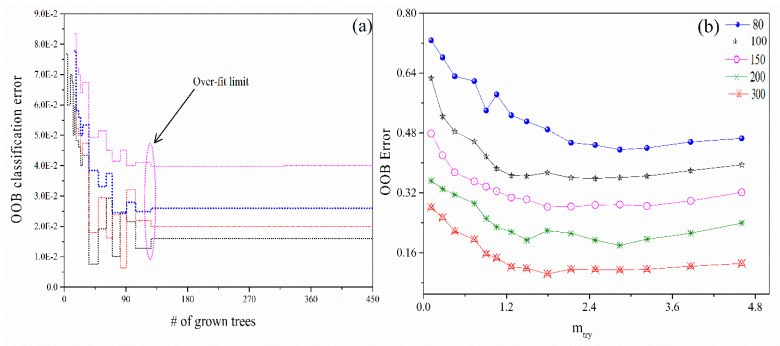
(**a**) The OOB classification error as a function of the grown number of trees in the RFT model (**b**) the OOB error as a function of the parameters (**n_tree_** and **m_try_**).

In RFT analysis, crucial parameters include the number of trees in the forest (**n_tree_**), the number of variables randomly selected (**m_try_**), and the OOB error for different **n_tree_** and **m_try_** settings. The **m_try_** parameter is a key feature in each split, introducing randomness by selecting attributes randomly. To enhance classification accuracy, the efficiency of the RFT simulation should be high. In assumption, increasing the number of trees (**n_tree_**) beyond an optimal point results in greater computational expense without significant improvement in results. In our study, the training sample includes P = 24,564 features (wavelengths). A subset of features **m_try_** was randomly selected under the condition **m_try_** ≪P. To build the model from the tested 450 trees, selecting the appropriate number of trees is a crucial step for running the RFT method. Figure 11a displays the OOB classification error as a function of the number of grown trees, indicating that the optimal number is 128 (~30 to 37% of the total from the original dataset). This number does not exhibit overfitting beyond that point and achieves a lower relative error, as highlighted by the elliptical dotted shape in the figure. Figure 11b illustrates the relationship between the OOB error and the **m_try_** parameter. The **n_tree_** parameter was set at 80, 100, 150, 200, and 300. The OOB classification error of the RFT model is relatively low when **n_tree_** is between 200 and 300, with the minimum classification OOB error occurring at 300 **n_tree_**.

### 3.4. RFT Classification and Validation

Different emission lines from the spectral range 280 to 400 nm were chosen as input data based on the nearly constant detector’s quantum efficiency with a better signal-to-noise ratio. A total of 400 LIBS emission spectra from alloy samples were utilized, with 320 optical spectra designated for the training set and 80 for the testing set. The emission lines including Al at 394.40, Zn at 334.50, Sn at 283.99, Si at 288.16, Cu at 324.75, and Pb at 283.31 nm were selected for RFT analysis due to their unsaturated signal intensities and minimal self-absorption. Spectral data sets were used to develop the RFT classification model, while test data sets were chosen as prediction samples to validate the model’s performance. Model performance is usually evaluated using n-fold cross-validation, but this approach often demands significant computation, reducing evaluation efficiency. As an alternative, the OOB data can be used to estimate classifier performance, requiring less computation once the OOB error rate is calculated. Table 4 presents a comparison between OOB estimation, including classification, sensitivity, accuracy, recall, and 10-fold cross-validation, including classification, accuracy, and RMSE, for sample classification. The classification accuracy of the OOB estimation was higher than that of the 10-fold cross-validation. The results from the 10-fold cross-validation were used as a benchmark to compare with the RFT results. OOB validation is valuable for RFT models due to its use of the bootstrap method for data selection. The findings reveal that the RFT method achieves a peak accuracy of ~98.89% based on the OOB classification, with an average prediction accuracy of 99.12% based on 10-fold cross-validation.

### 3.5. Quantitative Analysis

In this study, the CF-LIBS method is utilized to determine the chemical concentration (wt.%) of individual elements within the sample. Specifically, a one-line CF-LIBS technique is employed for sample quantification. Optically thin emission lines, Al (I) at 309.27 nm, Zn (I) at 577.21 nm, Sn (I) at 452.47 nm, Si (I) at 243.52 nm, Cu (I) at 510.55 nm, and Pb (I) at 373.99 nm, were used for CF-LIBS analysis. The normalized net integrated intensities of these selected emission lines were utilized for the analysis. The intensities were normalized by dividing with the net integrated intensity of Al (II) at 281.62 nm. The concentration of aluminum in different samples is not constant. However, we normalized the relative intensities of the emission lines to the intensity of Al (II) at 281.62 nm for each sample individually. In general, normalization minimizes the impact of experimental variations, such as differences in laser energy, plasma formation, or detector sensitivity. The concentration of neutral species in the sample is estimated by employing the Boltzmann equation [29,35].

(13) $$\bar{I_{\lambda }^{ki}}=F(\lambda )C^{\gamma }\left(\frac{A_{ki}g_{k}}{Z^{\gamma }(T)}\right){exp}^{\left(-\frac{E_{k}}{k_{B}T_{e}}\right)}$$

(14) $$Z^{\gamma }\left(T\right)=\sum_{i}g_{i}e^{-\frac{E_{i}}{k_{B}T_{e}}}$$

where $\bar{I_{\lambda }^{ki}}$ corresponds to the estimated integral line intensity, *T_e_* is the temperature of the electron plasma, *E_k_* (eV) is the upper-level energy, and *F* is the instrumental parameter depending on the optical efficiency of the detection arrangement, plasma volume, and its density. The instrumental parameter can be estimated through the normalization procedure for the elemental concentration. $Z^{\gamma }(T)$ represents partition function, $C^{\gamma }$ is the concentration of the neutral species, $g_{k}$ is the upper-level statistical weight, $A_{ki}(s^{-1}$) is the transition probability, and *k_B_* is the Boltzmann constant. The concentration of the ionized atoms $\left(C^{\gamma +1}\right)$ is estimated using the well-known Saha–Boltzmann equation [22]:

(15) $$\frac{C^{\alpha ,\gamma +1}}{C^{\alpha ,\gamma }}=\frac{6.04\times 10^{21}}{n_{e}}\sqrt[{3}]{T_{eV}}\frac{Z_{\alpha ,\gamma +1}}{Z_{\alpha ,\gamma }}exp[-\frac{E_{\alpha ,\gamma }}{k_{B}T}]$$

where $n_{e}({cm}^{-3})$ indicates number density, $Z_{\alpha ,\gamma +1}$ and $Z_{\alpha ,\gamma }$ are partition functions of the upper (γ+1) and lower (γ) charge states, respectively, $E_{\alpha ,\gamma }(eV)$ is the ionization energy, $C^{\gamma }$ and $C^{\alpha ,\gamma +1}$ are the concentrations of the neutral and ionized species, respectively. The atomic parameters that were exploited for the compositional analysis come from the NIST atomic spectral database [27]. The total composition (wt.%) is a sum of the composition of the neutral and ionized atomic species.

(16) $$C^{\alpha }=C^{\alpha ,\gamma }+C^{\alpha ,\gamma +1}$$

(17) $$Wt.\left(\%\right)=\frac{C^{\alpha }}{\sum {(C}^{\alpha ,\gamma }+C^{\alpha ,\gamma +1})}\times 100$$

For example, the CF-LIBS analysis for sample 6 yielded the following concentration values (wt.%): Al (54.60 ± 0.35), Cu (9.29 ± 0.15), Zn (9.13 ± 0.22), Sn (9.01 ± 0.39), Pb (8.97 ± 0.08), and Si (9.00 ± 0.13). These results exhibit a good agreement with the reference values for the prepared pelletized alloy samples.

**Figure 12:**
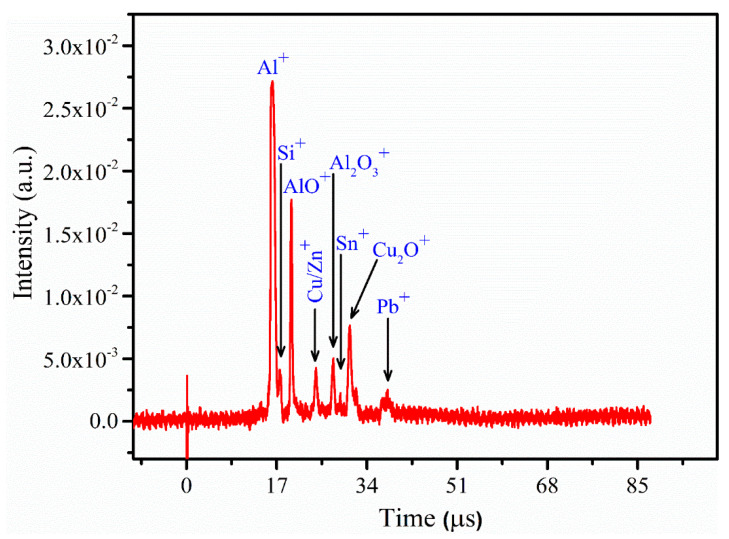
The mass spectrum of sample 6 obtained using LA-TOF-MS. The spectrum was recorded using a focusing lens with a focal length of 60 cm, a laser energy of ~5 mJ, and a focused beam radius at the target surface of roughly 0.5 (± 0.1) mm, all within a high vacuum environment of ~10^−6^ mbar.

In the LA-TOF-MS spectrometric analysis, all samples (1 to 9) under identical experimental conditions were examined. However, this study specifically presents the mass spectrum of the representative sample 6 as shown in Figure 12. To distinguish both atomic and molecular mass spectra, calibration was performed by establishing a linear relationship between the square root of the mass (√M) and the time of flight (T) of ions reaching the detector. The LA-TOF-MS spectrum illustrates the detection of various elements such as Al, Zn, Si, Sn, Cu, and Pb, each appearing on the time scale corresponding to their charge-to-mass ratio. Some of the elemental peaks were identified as molecular spectra of those elements, including AlO, Al_2_O_3_, and Cu_2_O. The presence of these molecular ions arises from the ionization process, wherein molecules may undergo fragmentation, resulting in the formation of charged fragments or ions. While certain molecules may remain intact as molecular ions due to their stable configurations, others may break apart into smaller charged fragments, including atomic ions. The composition and structure of the analyzed sample also influence the types of ions generated, with complex mixtures or molecules containing labile bonds being more susceptible to yielding both molecular and atomic ions. The various peaks in the LA-TOF-MS mass spectrum further demonstrate that Al is a predominant element in the matrix. Determination of the elemental composition is achieved by integrating the corresponding peaks and converting them to weight percentages. For instance, the estimated composition of sample 6 using LA-TOF-MS technique reveals Al (56.0 wt.%), Zn (8.7 wt.%), Sn (10.0 wt.%), Si (9.1 wt.%), Cu (8.0 wt.%), and Pb (8.2 wt.%), indicating a good agreement between the reference concentration and those calculated using CF-LIBS technique.

**Figure 13:**
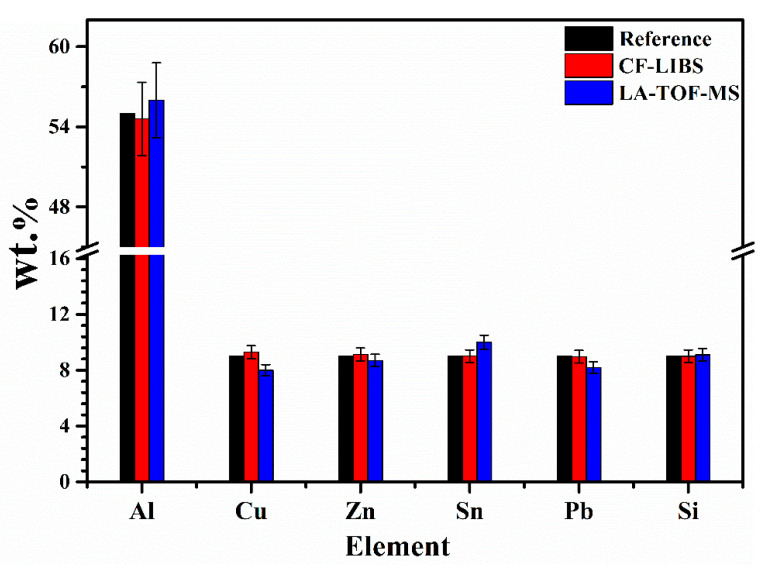
The chemical composition (wt.%) of Al, Zn, Sn, Si, Cu, and Pb in sample 6, estimated using CF-LIBS and LA-TOF-MS, is compared with the reference composition.

For the relative elemental analysis, the chemical composition (wt.%) of sample 6, determined using the CF-LIBS and LA-TOF-MS methods, is compared with the reference composition wt.% provided for the sample. This comparative analysis is visually represented in Figure 13. Both CF-LIBS and LA-TOF-MS results exhibit consistency with the reference composition within the margin of error. Aluminum emerges as the predominant element in sample 6, accounting for the largest fraction of the composition. Minor constituents, including Zn, Sn, Si, Cu, and Pb, are also detected, each contributing less than 10 wt.% to the overall composition. However, minor differences in the estimated compositions between LA-TOF-MS and CF-LIBS are observed and can be attributed to factors such as matrix effects, self-absorption effects in emission lines in CF-LIBS, and variations in detection sensitivity. While LA-TOF-MS directly quantifies ions, CF-LIBS relies on assumptions such as LTE and stoichiometric ablation, which may not always hold. Additionally, CF-LIBS may suffer from a lower SNR due to plasma continuum emission, whereas LA-TOF-MS typically provides higher precision in ion detection. Despite these challenges, the standard deviation calculated relative to the reference composition demonstrates strong agreement, indicating the reliability of both methods. To further improve the accuracy of CF-LIBS and LA-TOF-MS analyses, techniques such as advanced calibration models to address matrix effects, real-time plasma diagnostics to validate LTE conditions, and the use of internal standards can be employed. Optimization of laser parameters—such as beam energy, spot size, lens-to-plasma plume distance, pulse frequency, gate delay, and integration time—as well as standardized sample preparation can also reduce variability and enhance reproducibility. By mitigating these sources of error, inconsistencies between the methods can be minimized, ensuring more reliable and accurate elemental analysis.

## 4. Discussion

Numerous recent studies have highlighted various strategies for enhancing LIBS-based alloy analysis, yet our method offers a uniquely integrated and robust approach. For instance, in the regression contest [36], different teams applied preprocessing techniques, emission line selection, and advanced regression models such as PLS and neural networks to quantify Cr, Ni, Mn, and Mo in steel, reflecting the ongoing challenges in quantitative LIBS. Similarly, fs-LIBS studies [37] employed optimized experimental parameters and background correction methods (MF and SG filtering) to lower the detection limits for Mg, Cu, Mn, and Cr in aluminum alloys. Another study [38] improved LIBS detection limits for trace elements such as Be, Cu, Fe, Mg, Mn, and Si in aluminum alloy by using a high-resolution Echelle spectrometer and optimizing matrix effects and self-absorption. In contrast to these parameter-intensive approaches, our study uses a CF-LIBS method, carefully selecting non-resonant spectral lines and maintaining optically thin and LTE plasma conditions to ensure accurate quantification without the need for extensive preprocessing. Moreover, while the VAI-CON Chem system [39] demonstrated successful quasi-continuous LIBS analysis of molten steel at high temperatures and under pressure using a long-range optical system, it required a machine learning component for classification. The most comparative with our methodology is the study combining CF-LIBS and PLSR [40], which addressed chemical analysis without any sample pretreatment—a key strength for real-time industrial application. In that study, four alloy samples (three copper-based and one iron-based) containing major elements such as Al, Cr, Cu, Fe, Mn, Ni, and Zn, along with minor elements such as Sn and Si, were analyzed using both CF-LIBS and PLSR. The CF-LIBS method involved optimizing the temporal delay to ensure optically thin plasma under LTE, while PLSR was applied to selected spectral regions for robust calibration model development. The authors emphasized that CF-LIBS was effective for determining the elemental concentrations of a final alloy product, which then served as a reference for constructing PLS models that could rapidly monitor and assess the quality of newly produced materials of similar composition. Their combined approach reduced operational costs and facilitated real-time monitoring during and after the smelting process, highlighting LIBS’s potential in the metallurgical industry. However, unlike their reliance primarily on statistical regression models and time-resolved spectroscopy, our approach not only implements CF-LIBS for plasma parameter estimation and elemental quantification but also integrates LA-TOF-MS for cross-validation of results—providing higher analytical confidence. Furthermore, our use of an RFT technique based on spectral data offers an advanced machine-learning layer for functional alloy classification, achieving an striking accuracy of ~99.12% with 10-fold cross-validation. This broad LIBS–LA-TOF-MS–RFT workflow thus combines precise physical modeling, cross-verified mass spectrometric quantification, and robust classification, presenting an inclusive and scalable framework for alloy analysis and quality assurance in industrial settings.

The present study highlights both the strengths and limitations of CF-LIBS and LA-TOF-MS in the analysis of functional alloy materials, considering both qualitative and quantitative aspects. Although CF-LIBS is beneficial due to its calibration-free operation, it is also affected by matrix effects that can reduce accuracy in multi-component alloy systems. For instance, overlapping spectral lines in alloys such as Al–Cu–La–Ce may compromise the reliability of quantitative results. Additionally, its spatial resolution—typically above 40 µm—limits its ability to resolve microscale compositional features. In contrast, LA-TOF-MS offers higher mass resolution and sensitivity, with detection limits for many elements ranging from ng/g to µg/g. For trace elements such as Li and B, detection limits can reach as low as 5 µg/g, depending on the matrix. By comparison, CF-LIBS generally achieves detection limits in the 1–10 ppm range, although this can vary with laser energy, gating parameters, and sample composition. Both techniques require careful optimization of experimental parameters to ensure accuracy and reproducibility. Compared to conventional techniques such as ICP-OES and XRF, the proposed LIBS–LA-TOF-MS workflow offers significantly higher throughput and lower operational costs. ICP-OES typically involves labor-intensive sample digestion and the use of consumables, while XRF, although non-destructive, often lacks sensitivity for light elements. In contrast, the combined approach using LIBS and LA-TOF-MS with machine learning algorithms enables rapid, in situ analysis with minimal sample preparation, making it particularly valuable for real-time quality control of functional alloys.

## 5. Conclusions

In this work, pelletized alloy samples containing metals aluminum (Al), copper (Cu), lead (Pb), silicon (Si), tin (Sn), and zinc (Zn) were analyzed using laser-induced breakdown spectroscopy (LIBS) and laser ablation time-of-flight mass spectrometer (LA-TOF-MS). Laser-induced plasma was optimized, and measures were taken to address background interference and self-absorption of emission intensities within the temperature range of $9.0\times 10^{3}$ to $1.1\times 10^{4}$ K. Boltzmann plots utilizing non-resonant and self-absorption-free spectral lines of Zn and Sn were employed to determine plasma temperature, while electron number density was estimated using the *H_α_* emission line at 656.28 nm under local thermodynamic equilibrium and optically thin plasma conditions. A comparative elemental analysis of the representative sample 6 was performed using CF-LIBS and LA-TOF-MS techniques showing a good agreement between these techniques. Furthermore, the random forest technique (RFT) was used for rapid sample classification. The findings exhibit that this technique achieves a maximum accuracy of ~98.89% with OOB estimation. To validate the RFT results, 10-fold cross-validation was employed, yielding an average accuracy of ~99.12%. The results demonstrate that the integration of LIBS, LA-TOF-MS, and RFT offers a robust framework for the rapid assessment of functional metal alloys in advanced technological applications.

## Figures and Tables

**Table 1:** Chemical composition (wt.%) of Al, Cu, Pb, Si, Sn, and Zn in pelletized alloy samples.

*Sample*	*Element Chemical Composition (wt.%)*					
	*Aluminum*	*Copper*	*Lead*	*Silicon*	*Tin*	*Zinc*
1	99.5	0.1	0.1	0.1	0.1	0.1
2	97.5	0.5	0.5	0.5	0.5	0.5
3	95	1	1	1	1	1
4	85	3	3	3	3	3
5	70	6	6	6	6	6
6	55	9	9	9	9	9
7	40	12	12	12	12	12
8	25	15	15	15	15	15
9	5	19	19	19	19	19

**Table 2:** Spectroscopic parameters of the neutral lines of Sn and Zn to create the Boltzmann plots [27].

*Wavelength λ* (nm)	*Transition*		*Transition Probability* $A_{k}\;(10^{7}s^{-1}$)	*Upper Energy Level* $E_{k}$ $({cm}^{-1}$)	*Statistical Weight* (2J_k_ + 1)		*Accuracy*
	*Upper Level*	*Lower Level*			$g_{k}$	$g_{i}$	
Tin (Sn):							
300.91	5*p*6*s* ^3^*P*_1_ → 5*p*^2 3^*P*_1_		3.8	34,914.282	3	3	*D*
303.41	5*p*6*s* ^3^*P*_0_ → 5*p*^2 3^*P*_1_		20	34,640.758	1	3	
314.18	5*p*5*d* ^3^*P*_1_ → 5*p*^2 1^*S*_0_		1.9	48,981.934	3	1	
333.06	5*p*6*s* ^3^*P*_2_ → 5*p*^2 1^*D*_2_		2.0	38,628.876	5	5	
380.10	5*p*6*s* ^3^*P*_1_ → 5*p*^2 1^*D*_2_		2.8	34,914.282	3	5	
452.47	5*p*6*s* ^1^*P*_1_ → 5*p*^2 1^*S*_0_		2.6	39,257.053	3	1	
Zinc (Zn):							
328.23	4*s*4*d* ^3^*D*_1_ → 4*s*4*p* ^3^*P*_0_		9.0	62,768.7462	3	1	*B*
330.25	4*s*4*d* ^3^*D*_2_ → 4*s*4*p* ^3^*P*_1_		12	62,772.0144	5	3	
334.50	4*s*4*d* ^3^*D*_3_ → 4*s*4*p* ^3^*P*_2_		17	62,776.9809	7	5	
468.01	4*s*5*s* ^3^*S*_1_ → 4*s*4*p* ^3^*P*_0_		1.6	53,672.2398	3	1	*NR*
472.21	4*s*5*s* ^3^*S*_1_ → 4*s*4*p* ^3^*P*_1_		4.6	53,672.2398	3	3	
481.05	4*s*5*s* ^3^*S*_1_ → 4*s*4*p* ^3^*P*_2_		7.0	53,672.2398	3	5	
577.21	4*s*7*p* ^3^*P*_2_ → 4*s*5*s* ^3^*S*_1_		0.08	70,992.3040	5	3	
636.23	4*s*4*d* ^1^*D*_2_ → 4*s*4*p* ^1^*P*_1_		4.7	62,458.5323	5	3	*C*

**Table 3:** Calculated [27] and observed intensity ratios used to validate the optically thin plasma criterion from the spectrum of sample 6.

*Element*	*Wavelength* *λ* (nm)	*A_k_g_k_* (10^8^) s^−1^	*E_k_* (eV)	*T_e_* (eV)	*Calculated*	*Observed*
*Pb (I)*	368.3	1.37	4.33	0.89	0.67	0.68
	283.3	1.50	4.37			
*Si (I)*	250.7	2.74	4.95		0.42	0.41
	288.2	6.51	5.08			
*Zn (I)*	468.01	0.16	6.65		2.85	2.73
	472.22	0.46	6.65			

**Table 4:** Comparison of RFT classification and accuracy built on OOB predictions and 10-fold cross-validation for all samples.

*Sample Type*	*RFT Results Based on OOB*				*RFT Classification Based on 10-Fold* *(Cross-Validation)*		
	*Classification*	*Sensitivity*	*Accuracy*	*Recall*	*Classification*	*Accuracy*	*RMSE*
1	100% (1.00~10/10)	0.9952	0.9976	96.91%	93.6	0.9476	1.228
2		0.9865	0.9971	91.67%	95	0.8971	0.851
3		0.9976	0.9874	98.70%	94	0.8879	0.899
4		0.9874	0.9975	95.23%	92	0.9677	0.978
5		0.9471	0.9576	91.92%	88	0.9576	1.199
6		0.9972	0.9878	92.78%	94.5	0.9878	0.965
7		0.9877	0.9779	94.92%	98	1.2778	0.981
8		1.0000	0.9972	98.80%	99.6	0.9972	0.982
9		0.9979	1.0000	97.23%	100	0.9998	1.309

## Data Availability

The raw data supporting the conclusions of this article will be made available by the authors on request.
